# Effects of Inactivation of the Periaqueductal Gray on Song Production in Testosterone-Treated Male Canaries (*Serinus canaria*)

**DOI:** 10.1523/ENEURO.0048-20.2020

**Published:** 2020-08-14

**Authors:** Chelsea M. Haakenson, Jacques Balthazart, Gregory F. Ball

**Affiliations:** 1 Program in Neuroscience and Cognitive Science, Department of Psychology, University of Maryland, College Park, MD 20742,; 2 Laboratory of Behavioral Neuroendocrinology, GIGA Neurosciences, University of Liege, 15 Avenue Hippocrate, 4000, Liege, Belgium

**Keywords:** motivation, periaqueductal gray, singing behavior, songbird

## Abstract

Male canaries (*Serinus canaria*) display seasonal changes in the motivation to sing which have been found to be dependent on the action of testosterone (T). During the breeding season when T is high, males sing at a higher rate compared with males with low T. The effect of T on song rate is known to be mediated by the medial preoptic nucleus (POM); however, it is unclear how T signaling in POM impacts song production. One potential mechanism is via modulation of dopaminergic input into song control nuclei by the periaqueductal gray (PAG). In order to test the role of PAG in T-mediated song production, we treated male canaries with peripheral T implants and implanted a guide cannula targeting the PAG. Through this guide cannula, we transiently inactivated PAG with injections of the GABA_A_ agonist, muscimol. Each bird received multiple infusions of both muscimol and saline with a 48-h washout period between treatments. The order of injection type was randomized and counterbalanced between individuals. Muscimol infusion into the PAG, but not nearby regions, increased the latency to sing post-injection. These results support the hypothesis that PAG is involved in the production of song, potentially mediating the motivation to sing or alternatively interfering with the pre-motor activity of nucleus RA. Other song features were however not affected.

## Visual Abstract

**Figure:**
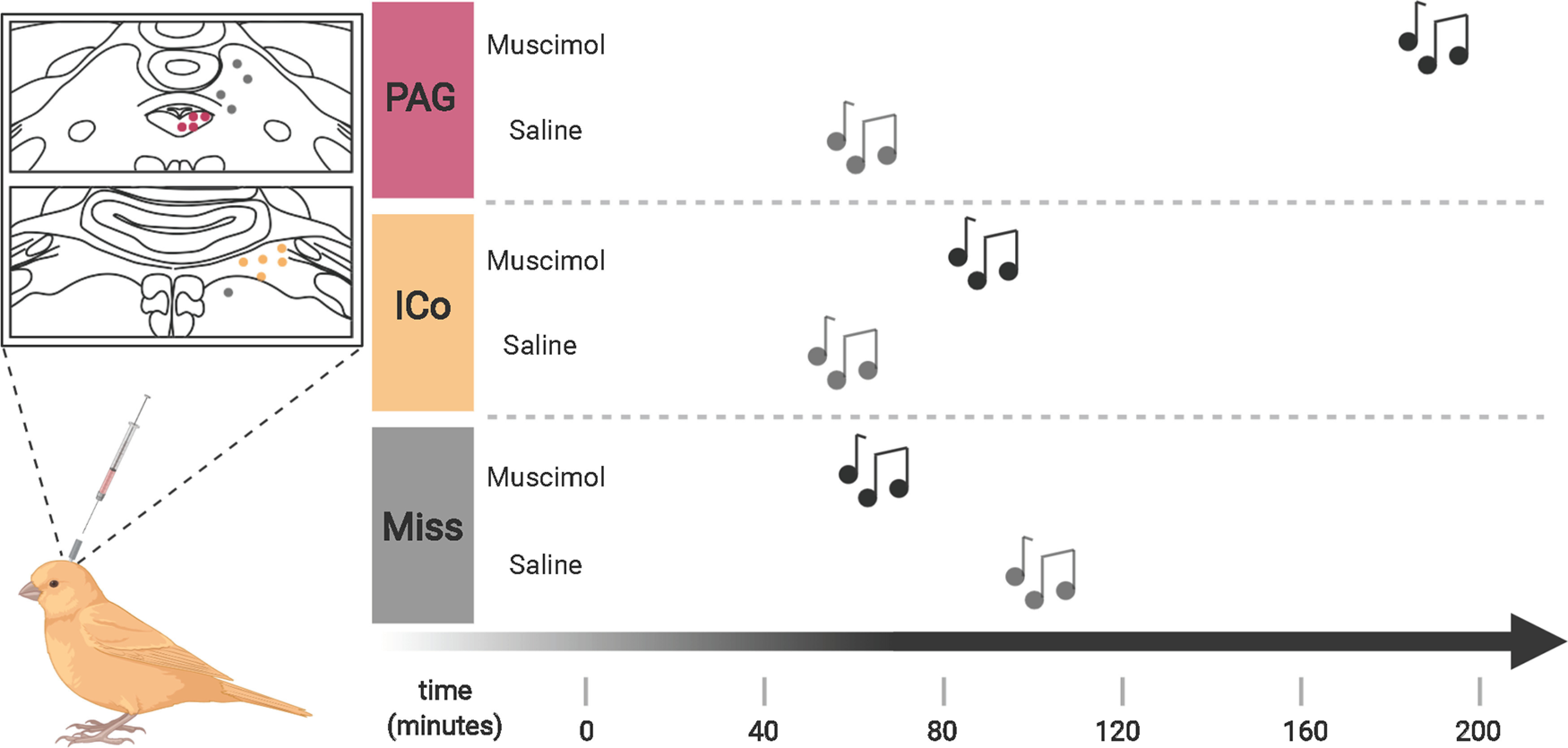

## Significance Statement

Communication is essential for social species relying on coordinated behavior for survival and reproduction. However, the neural mechanisms underlying the motivation to engage in vocal communication are currently unknown. Here, we show that inhibition of the periaqueductal gray (PAG) increases the latency for male canaries to sing but does not influence features of song quality once they resume singing. These results indicate that the PAG is involved in regulating the motor initiation or the underlying motivation of the complex, learned behavior of singing but not of innate vocalizations, such as calls. Our findings suggest that the PAG is likely involved in transmitting preoptic signals to the song control system, probably via dopaminergic projections from this region to song control nuclei.

## Introduction

For social species, communication between individuals is essential for forming bonds and promoting survival. Without the ability to communicate, the benefits of living in a social group, such as coordinated food gathering, collective defense of self and territory, mating, and caring for young, are unattainable. However, having the ability to communicate verbally is useless if one does not actually do so. Therefore, individuals that live in social groups must be motivated to engage in vocal communication. Currently, the neural circuits and molecular mechanisms underlying this motivation are largely unknown.

Songbirds are an ideal model for addressing this problem, as they rely on vocal communication to relay biologically significant information, and have a vocal learning process with many parallels to that of humans (Doupe and Kuhl, 1999; Kuhl, 2000, 2003; Konishi, 2004). While song can have a variety of functions, one of its key utilities is in mate attraction (Catchpole and Slater, 2008). Appropriately, in seasonally breeding songbird species such as the canary (*Serinus canaria*), the motivation to sing increases during the breeding season, when attracting a mate is particularly important (Voigt and Leitner, 2008). At this time, males also have increased plasma testosterone (T; Ball et al., 2002; Leitner et al., 2003; Ball and Balthazart, 2017). It is this increase in T that leads to a heightened motivation to sing, in addition to a variety of other effects on song quality (Ball et al., 2003).

This effect of T on the motivation to sing appears to occur via activity in the medial preoptic nucleus (POM), since implantation of T specifically in this region in castrated male canaries increases the number of songs per minute and the percentage of time spent singing (Alward et al., 2013). In addition, partially lesioning the POM has the inverse effect, causing male European starlings (*Sturnus vulgaris*) to sing less frequently (Riters and Ball, 1999; Alger et al., 2009). However, it is not yet known how T in POM can induce such changes in song behavior.

One potential pathway for transmission of T-modulated activity in POM to the song control system is via the periaqueductal gray (PAG). There are reciprocal projections between PAG and POM (Riters and Alger, 2004). In addition, PAG sends dopaminergic projections to several nuclei in the song control system, HVC, the robust nucleus of the arcopallium (RA), and area X (Lewis et al., 1981; Appeltants et al., 2000, 2002; Castelino et al., 2007). Neural activity in PAG also appears to be tied to activity in POM, as European starlings with lesions to POM have decreased expression of the immediate early gene ZENK in the PAG (Alger et al., 2009). Furthermore, DOPAC, a metabolite of dopamine, in PAG has been found to correlate with song production (Heimovics et al., 2011).

In the present study, we examine the role of PAG in the control of singing. We castrated male canaries and implanted them with SILASTIC implants that continuously release T, ensuring high singing rates. We then implanted guide cannulas to target the PAG for neurochemical manipulation in these males. We conducted six trials per individual, alternating infusion of saline (vehicle) or the GABA_A_ agonist muscimol into this brain region. We hypothesized that PAG transmits to the song system T-modulated activity from POM and accordingly sends motivational cues to song control nuclei. Therefore, we predicted that during trials in which PAG was transiently inactivated, males would either refrain from singing for some time following infusion or sing less frequently than in control trials. Furthermore, we predicted that this effect would be limited to song, since T-modulated changes in vocal behavior are limited to song, which is learned, and do not affect calls, which are innate.

## Materials and Methods

All animal procedures were performed in accordance with the University of Maryland, College Park animal care committee’s regulations.

### Experimental animals and pre-experimental manipulations

Fourteen adult male canaries (*S. canaria*) of the American Singer strain were obtained from a local breeder (Maryland Exotic Birds). Upon arrival, birds were housed in grouped aviaries on a short-day photoperiod (8/16 h light/dark; 8L:16D) to induce photosensitivity (Nicholls and Storey, 1977). All animals were provided with *ad libitum* food and water. Male birds were castrated under anesthesia (Forane isoflurane, Baxter; Isotex four anesthesia machine, Surgivet) through an incision between the last two ribs on each side. Birds were allowed to recover in their home cage for six weeks to allow adequate time for any residual physiological or behavioral effects of endogenous T to clear.

### Stereotaxic implantation

Birds were anesthetized using isoflurane gas and placed in a stereotaxic apparatus modified for use in small birds. Beaks were placed in a custom, 3D-printed beak holder placed at 45° below the horizontal axis of the apparatus. Each bird received a unilateral guide cannula (26 gauge, C315GMNSPC, Plastics One) implant targeting PAG. In order to target PAG without puncturing the ventricle and inducing bleeding, we oriented the stereotaxic arm at a 40° angle to the right from vertical and then used the following stereotaxic coordinates: dorsoventral: –7.1 mm from the dorsal surface of the brain; anterior-posterior: 0 mm from the most rostral tip of the cerebellum; medial-lateral: 4.5 mm to the right from midline. The cannula was lowered to the target coordinates, and dental cement was applied around the implant. The skin was sutured around the implant. At the time of stereotaxic surgery, males were also implanted subcutaneously with a 12-mm-long SILASTIC implant (Dow Corning; internal diameter, 0.76 mm; external diameter, 1.65 mm) packed with 10 mm of T to standardize circulating T levels. Following recovery from surgery, males were transferred to individual sound-attenuating chambers (41 × 48 × 51 cm) set to 14L:10D to simulate breeding photoperiods.

### Microinfusion procedures

In the week following surgery, birds were handled daily and the dummy injectors were removed and reattached so that the birds would habituate to experimental manipulations. After a male had been observed to sing at least 2 d in a row, he underwent a mock infusion session to acclimate him to the infusion procedure, in which saline was infused through the internal cannula in the same protocol as later test infusions. Test infusions began 2 d after the acclimation infusion. Each bird underwent six experimental infusion sessions: three sessions with 0.2 μl 0.9% saline (vehicle) and three sessions with muscimol in 0.9% saline (0.5 μg/0.2 μl solution, catalog #0289, Tocris). Infusions alternated between vehicle and drug, but the starting infusion type was randomized and counterbalanced across individuals. Infusion sessions were separated by at least 48 h to allow for drug washout. During infusions, the dummy injector was replaced by an internal cannula (33 gauge, C315IMNSPC, Plastics One) connected to a Hamilton microliter syringe via a polyethylene tube. The syringe was loaded into a syringe pump (KDS 220, KD Scientific) programmed to deliver treatment (vehicle or drug) at a rate of 0.1 μl/min for 2 min, for a total volume of 0.2 μl. Following infusion, the internal cannula was held in place for an additional minute to allow for diffusion and to avoid reflux of the solution. Birds were then returned to their home chamber. Chambers contained a combination microphone/camera (Mini Spy HD 1000TVL, TPEKKA) connected to a computer running DVRserver (V6.33b; Mammoth Technologies) designed for real-time video and audio surveillance. Video and audio were recorded for the remainder of the day following infusions.

### Behavior quantification

Videos for each day were downloaded and converted into two filetypes: AVI files for video viewing and WAV files for audio analysis. In order to quantify the latency to sing, we used two independent measures. First, videos were watched from the time a bird was returned to the chamber after drug infusion until the bird was observed singing. The difference in time between infusion and singing was recorded. Audio files were independently inspected in Adobe Audition, and the time between the sound of the bird being returned to the chamber and the start of singing was recorded. These two measures were then compared with ensure that the recorded latency to sing was accurate. In two instances where there were differences between the latency recorded from videos or from audio files, another investigator analyzed the corresponding files to determine which measure was correct and ultimately a consensus was reached on the true time when the first song occurred. For measures of song quality, we used Audition to clip audio files to be 1 h in length, starting at the bird’s first song post-infusion. These files were analyzed using Avisoft (SASlab Pro). Songs were defined as bouts of vocalizations longer than or equal to 1 s in duration and separated by 500 ms of silence (Alward et al., 2013). One bird was excluded from any analysis of song quality, because of high background noise from the chamber fan. In order to measure the number of calls, experimenters watched video recordings of the hour following return to the chamber following infusions. For each minute of the hour, experimenters counted the number of calls that occurred during that minute, and all counts were summed at the end to get the number of calls that occurred during the entire hour. The same procedure was used to quantify perch hops, as a measure of general activity following infusion, for 20 min of video following return to the chamber. For all behavior quantification procedures, experimenters were blind to treatment and cannula placement.

### Verification of cannula targets

After birds completed all six test microinfusion sessions, we performed a final microinfusion of 0.2-μl fluorescent-conjugated muscimol (Muscimol BODIPY TMR-X Conjugate, catalog #M23400, ThermoFisher) according to the procedure described above. We allowed 30 min for muscimol diffusion before extracting brains and flash freezing them on dry ice. Brains were stored at −80°C. Brain tissue was sectioned with a cryostat (Microm HM 500 OM) at 50 μm in the coronal plane and directly mounted on slides. We then visualized the spread of fluorescent muscimol and the location of tracts created by the guide cannula to determine whether the cannula was accurately targeting PAG for drug infusion.

### Data analysis

We analyzed latency, song measures, and call data using repeated-measures ANOVA, with cannula placement and treatment (muscimol, saline) as factors. In addition, we performed estimation based on confidence intervals (CIs) using the Data Analysis using Bootstrap-Coupled Estimation (*dabestr*) package, written for use in the R programming language (Ho et al., 2019). For song measures, which encompassed singing behavior in the hour after the bird began singing, we combined data from multiple songs to create a single value for each acoustic feature, averaged across all vocalizations in the hour of audio quantified for each individual and trial. Total number of songs was calculated by adding the number of vocalizations that met our criteria for song. Time spent singing was calculated by adding the duration of each of these songs. For measures that concerned each individual song (song duration, interval between songs, number of song elements, energy, peak to peak amplitude, root mean square (RMS) amplitude, entropy, fundamental frequency, bandwidth, peak amplitude, and peak frequency), we averaged the values of each measure across the songs sung within the 1-h song file for a given trial. These measures were chosen to quantify a range of song features including stereotypy, volume, and frequency range.

## Results

**Figure 1.**
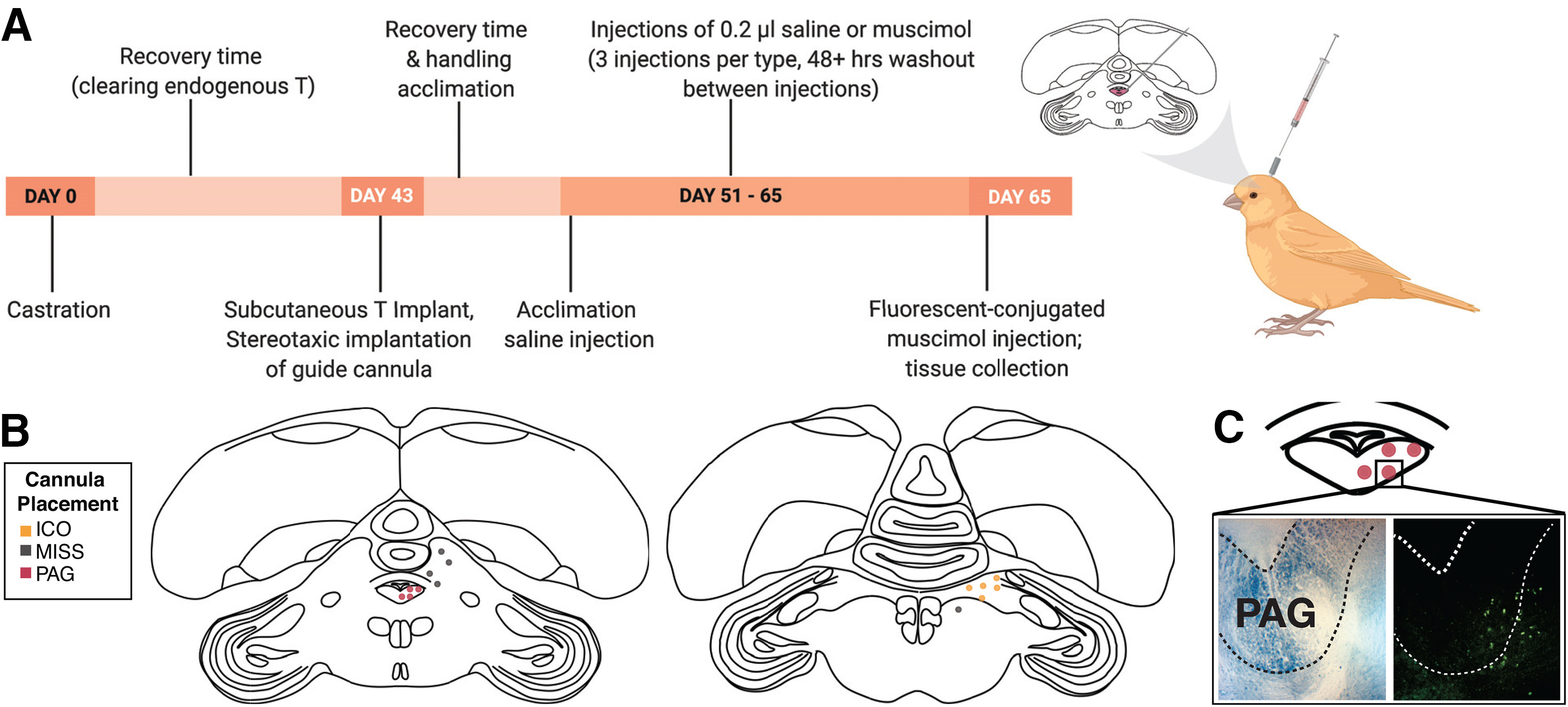
Experimental procedures. ***A***, Timeline of experimental procedures (left) and image of cannula trajectory (right) created with BioRender. ***B***, Coronal sections demonstrating final placement of cannula targets after surgery, four in PAG, five in ICo, and five misses. ***C***, Example images of muscimol spread for an individual where cannula placement was on the lateral edge of PAG. Left is a Nissl brightfield photomicrograph showing the location of PAG and right is the corresponding fluorescent photomicrograph.

Because of variation in accuracy of stereotaxic implantation of guide cannulas, subjects were categorized in three groups based on the location: PAG, intercollicular nucleus (ICo), and misses (Fig. 1). Five males were categorized in the ICo group, five in the miss group, and four in the PAG group. The fluorescent muscimol spread an average of 533 μm in the ventral-medial direction away from the end of the guide cannula tract and an average of 225 μm in the perpendicular direction. In individuals where the guide cannulas targeted PAG, this spread was sufficient to cover at least some portion of the contralateral side, in addition to affecting the hemisphere containing the guide cannula. When guide cannulas targeted regions that are located more laterally (ICo and misses), this spread only affected the hemisphere ipsilateral to the guide cannula. To determine whether muscimol diffused into the aqueduct and was transported to other brain regions via the ventricular system, we assessed fluorescence around the aqueduct and across brain regions in the telencephalon and diencephalon. We did not observe fluorescence above standard autofluorescence in any brain regions outside the mesencephalon near cannula tracts.

### Song latency

**Figure 2.**
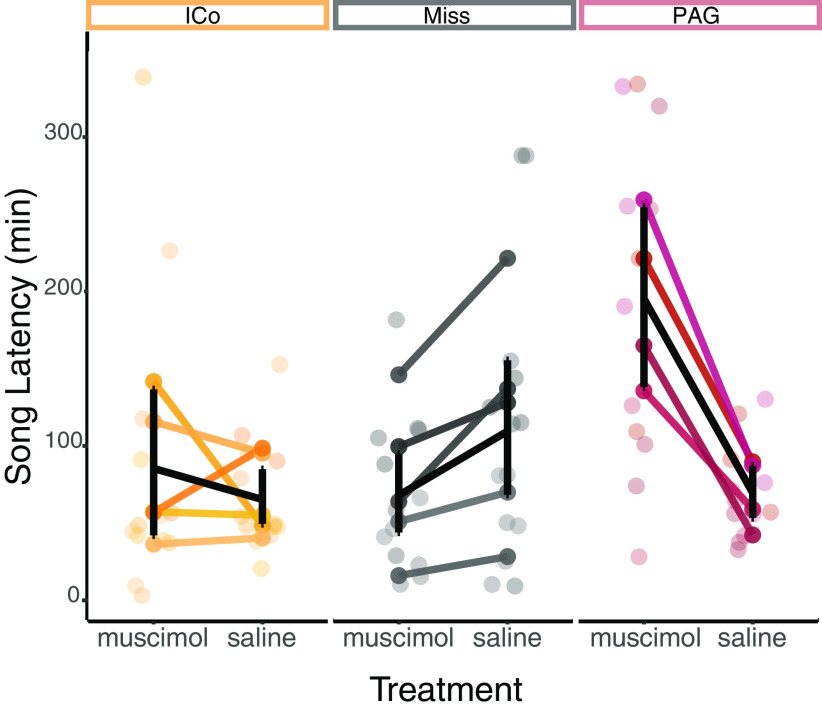
Change in latency to begin singing post-infusion. Differences in latency for each individual across trial type. Each line connects the average latency to sing following muscimol infusion to the average latency following saline infusion for an individual bird. Black lines indicate summary statistics (mean and SEM), while colored lines indicate the average latency for each individual bird. Individual points indicate the latency for each single trial. The three sections of the graph represent cannula placement (ICo, miss, or PAG). In order to determine whether an ANOVA was appropriate to determine differences between treatments, we tested the assumption of homoscedasticity (Extended Data Fig. 2-1*A*). To further assess the differences between groups and treatments, we used bootstrapped estimation statistics of 95% CIs (Extended Data Fig. 2-1*B*).

There was a significant difference in latency to begin singing between muscimol and saline trials (*F*_(1,11)_ = 5.775, *p* = 0.035, η^2^ = 0.046) and a significant interaction between cannula placement and treatment (*F*_(2,11)_ = 19.222, *p* < 0.001, η^2^ = 0.303; Fig. 2). There was no significant main effect of cannula placement alone on latency to sing (*F*_(2,11)_ = 1.873, *p* = 0.200, η^2^ = 0.143). The effect of treatment was driven by differences in the group with guide cannulas targeting PAG. In birds with cannulas targeting PAG, there was a large increase in time to sing post-infusion for muscimol trials compared with saline trials [mean muscimol latency minus saline latency (ΔL) = 125.75 min, SE = 13.50], while differences in latency in birds with cannula targeting ICo (ΔL = 20.10 min, SE = 14.34) and in birds with cannula targets classified as misses (ΔL = −41.50 min, SE = 20.43) were smaller. We found a similar pattern of results by performing estimation statistics with the *dabestr* R package. We created 5000 bootstrapped sample distributions representing the difference in latency to sing for muscimol trials minus saline trials, such that a positive estimation would indicate a larger latency to sing following muscimol infusions compared with saline infusions. The PAG group had a 95% CI that indicated a larger latency to sing following muscimol trials compared with saline trials (126 min, 95% CI [71.8; 184]). The 95% CI for the ICo group (28 min, 95% CI [−3.21;113]) included 0, indicating that there is likely no difference in latency to sing between muscimol and saline trials. The Miss group’s 95% CI (−38.8 min, 95% CI [−99.5; −9.51]) was close to zero, but did not include zero, indicating a small increase in latency to sing for saline trials.

10.1523/ENEURO.0048-20.2020.f2-1 Extended Data Figure 2-1 Plots supporting statistical analysis of latency data. ***A***, Plot showing homoscedasticity assumption for ANOVA. Distribution of residuals are plotted against treatment and brain region targeted. ***B***, Plot of CIs of differences between muscimol trials and saline trials bootstrapped 5000 times. Download Figure 2-1, EPS file.

### Song measures

**Figure 3.**
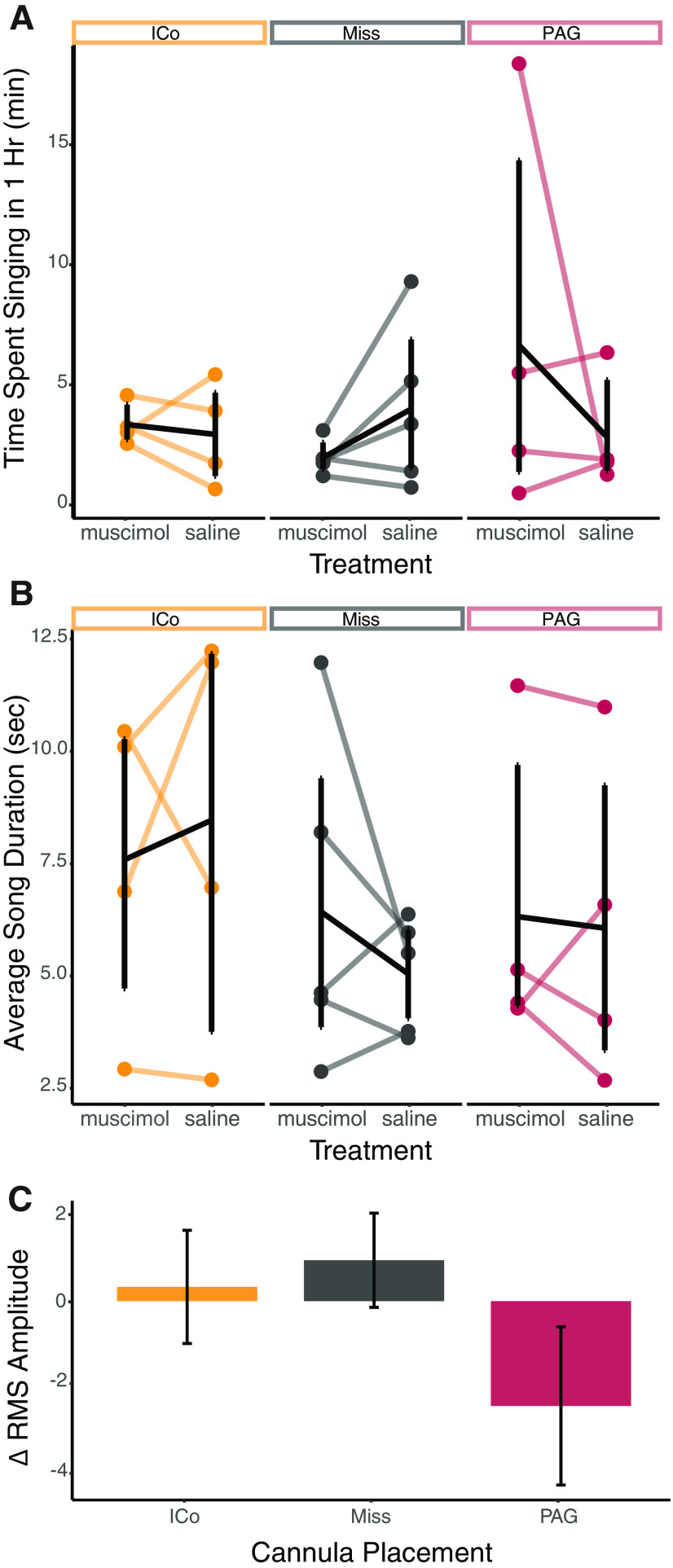
Example song quality measurements. ***A***, The amount of time in minutes spent singing 1 h after singing began. There were no significant differences between treatments or cannula placement. The three sections of the graph represent cannula placement (ICo, miss, or PAG). ***B***, The average duration of songs in seconds. There were no significant differences between treatments or cannula placement. ***C***, Average change in RMS amplitude between treatments (muscimol minus saline). There was a significant interaction between treatment and cannula placement but no main effect. Each line connects the average data following muscimol infusion to the average data following saline infusion. Black lines indicate summary statistics (mean and SEM), while colored lines indicate the average data for each individual bird.

In the hour after birds began singing, we did not find any significant differences between treatments (saline or muscimol) or between cannula placements for the amount of time spent singing (total number of songs, total amount of time spent singing) or measures of song quality (song duration, interval between songs, energy, number of elements, peak to peak frequency, fundamental frequency, entropy, bandwidth, or amplitude; Fig. 3; for results of statistical analyses, see Table 1). We did observe a significant interaction between treatment and placement for RMS amplitude (*F*_(2,10)_ = 4.828, *p* = 0.0341). However, the lack of significant main effects of cannula placement (*F*_(2,10)_ = 0.485, *p* = 0.629) or treatment (*F*_(1,10)_ = 0.312, *p* = 0.589) and the small effect size (η^2^ = 0.012) suggests that this may be a spurious effect. Furthermore, the 95% CI of bootstrapped differences between muscimol and saline trials included zero in all groups, supporting the idea that this is not a real effect (PAG: −2.43 RMS power, 95% CI [−5.19; 0.123]; ICo: 0.528 RMS power, 95% CI [−0.002; 1.52]; Miss: 0.796 RMS power, 95% CI [−0.942; 2.31]).

**Table 1:** Results of two-way repeated measures ANOVA of song quality measures

Song measure	Factor	df	*F*	*p*	Estimation statistics difference (muscimol – saline)		
Number of songs	Cannula placement Treatment interaction	2 1 2	0.328 0.005 1.855	0.728 0.944 0.206	ICo Miss PAG	8.5 –23.4 25.2	95 CI [–6.67; 22.3] 95 CI [–9.83; 140] 95 CI [–9.83; 140]
Time spentsinging	Cannula placement Treatment interaction	2 1 2	0.574 0.130 1.359	0.581 0.726 0.301	ICo Miss PAG	24.6 –117 230	95 CI [–58.9; 89.3] 95 CI [–229; –20.2] 95 CI [–56.5; 1240]
Average songduration	Cannula placement Treatment interaction	2 1 2	0.662 0.164 0.626	0.537 0.694 0.554	ICo Miss PAG	–0.88 0.089 0.256	95 CI [–3.34; 1.35] 95 CI [–1.06; 1.55] 95 CI [–1.56; 1.77]
Average intersonginterval	Cannula placement Treatment interaction	2 1 2	0.757 0.0 0.2	0.494 0.985 0.822	ICo Miss PAG	–13.6 9.61 39	95 CI [–153; 43.4] 95 CI [–147; 166] 95 CI [–180; 203]
Average songelements	Cannula placement Treatment interaction	2 1 2	0.747 0.407 0.354	0.499 0.538 0.710	ICo Miss PAG	1 –0.661 1.3	95 CI [–1.78; 6.86] 95 CI [–14; 14] 95 CI [–1.89; 6.2]
Average energy	Cannula placement	2	0.607	0.564	ICo	0.0002	95 CI [0.00004; 0.0006]
	Treatment interaction	1	0.001	0.989	Miss	–0.000008	95 CI [–0.0009; 0.0001]
		2	2.541	0.128	PAG	–0.0003	95 CI [–0.0009; 0.0001]
Average peak topeak amplitude	Cannula placement Treatment interaction	2 1 2	1.12 0.753 2.616	0.372 0.411 0.134	ICo Miss PAG	0.014 0.003 –0.037	95 CI [0.001; 0.038] 95 CI [–0.014; 0.026] 95 CI [–0.083; 0.008]
Average RMS	Cannula placement Treatment interaction	2 1 2	0.485 0.312 4.828	0.629 0.589 0.034*	ICo Miss PAG	0.528 0.796 –2.43	95 CI [–0.002; 1.52] 95 CI [–0.942; 2.31] 95 CI [–5.19; 0.123]
Average entropy	Cannula placement	2	0.007	0.993	ICo	0.0009	95 CI [–0.010; 0.008]
	Treatment interaction	1	1.683	0.224	Miss	–0.0009	95 CI [–0.007; 0.002]
		2	0.171	0.845	PAG	0.0004	95 CI [–0.014; 0.012]
Max. fundamentalfrequency	Cannula placement Treatment interaction	2 1 2	0.385 1.274 0.014	0.690 0.285 0.986	ICo Miss PAG	–52.4 –80 –44.3	95 CI [–363; 225] 95 CI [–221; 85.2] 95 CI [–613; 614]
Max. bandwidth	Cannula placement Treatment interaction	2 1 2	0.343 0.084 0.747	0.718 0.778 0.498	ICo Miss PAG	110 –43.4 –79.4	95 CI [–129; 495] 95 CI [–286; 37] 95 CI [–348; 271]
Max. peakamplitude	Cannula placement Treatment interaction	2 1 2	0.641 0.512 2.214	0.547 0.491 0.160	ICo Miss PAG	0.332 0.15 –2.88	95 CI [–1.94; 2.43] 95 CI [–3.64; 2.84] 95 CI [–6.2; –0.045]
Max. peakfrequency	Cannula placement Treatment interaction	2 1 2	1.033 1.184 0.236	0.391 0.302 0.794	ICo Miss PAG	7.83 –182 –148	95 CI [–217; 149] 95 CI [–749; 45.9] 95 CI [–730; 492]

### Calls

**Figure 4.**
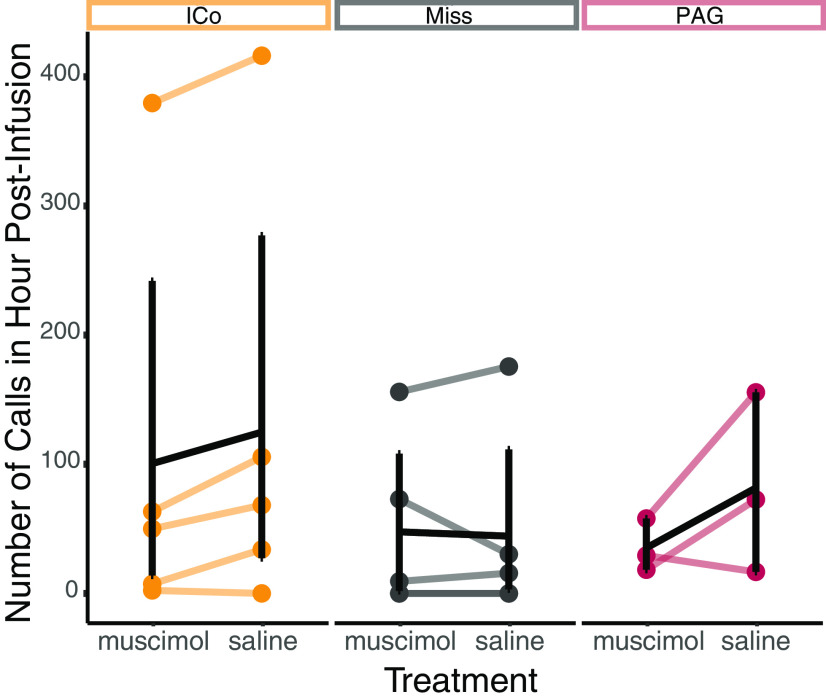
There were no significant differences in call rate in the hour following infusion. Difference between muscimol and saline trials (average number of calls following muscimol infusion minus average number of calls following saline infusion) for individual birds. Colors represent cannula placement. Each line connects the average call rate following muscimol infusion to the average call rate following saline infusion. Black lines indicate summary statistics (mean and SEM), while colored lines indicate the average data for each individual bird.

We also quantified the number of calls birds made in the hour immediately following infusion and found no significant differences between treatments (*F*_(1,11)_ = 4.783, *p* = 0.054), cannula placements (*F*_(2,11)_ = 0.468, *p* = 0.639), or an interaction between the two (*F*_(2,11)_ = 2.549, *p* = 0.127; Fig. 4). Likewise, 95% CIs of the difference between muscimol and saline trials included zero for all three cannula placements (PAG: −53.9 calls, 95% CI [−140; 0.75], ICo: −24.3 calls, 95% CI [−92.3;53], Miss: 4.31 calls, 95% CI [−18.2; 44.2]).

### General activity

We did not find a difference in the number of perch hops following infusion between cannula placements (*F*_(2,11)_ = 0.546, *p* = 0.594), between saline and muscimol trials (*F*_(1,11)_ = 0.228, *p* = 0.643), or an interaction between placement and treatment for any cannula placement. (*F*_(2,11)_ = 0.942, *p* = 0.419). Likewise, the 95% CIs of difference between muscimol and saline trials included zero for all conditions (PAG: −19.3 perch hops, 95 CI [−68.9; 0.1], ICo: −5.6 perch hops, 95 CI [−27.1; 3.4], miss: 11.3 perch hops, 95 CI [−8.27; 37.2]).

## Discussion

This study found that transiently inactivating the PAG increases the latency to sing in castrated male canaries treated with exogenous T. Since muscimol infusion into this region increased the latency to sing, while having no effect on other aspects of song production, PAG seems to be specifically involved in the initiation of singing behavior. This could be because of PAG conveying cues from the POM to the song control system. The interpretation of these results depends critically on the assumed duration of muscimol infused into the PAG. Based on past pharmacological studies, it is likely that the dose of 0.5-μg muscimol we infused in the PAG activated GABA_A_ receptors for a duration of one or 2 h. Indeed a previous study found that an intraperitoneal injection of muscimol produced an analgesia in the hot plate test that was fully effective for 40 min and was already decreasing at 60 min, with an extrapolated return to baseline before 2 h after injection (Sawynok and Labella, 1982). Using an approach more similar to the present work, muscimol infused into the preoptic area decreased the lordosis quotient of female rats at 10 and, to a lower extent, 30 min after infusion, but the behavior returned to baseline at 60 min. A similar time course was observed for the stimulation of lordosis by muscimol injected into the medial hypothalamus (McCarthy et al., 1990). These durations of action depend on the doses that are injected but studies combining doses and duration are rare and, to our knowledge, do not exist for brain injections. Based on these data, it seems likely that our injections of a low dose of muscimol modulated GABA_A_ activity for a duration of about 1–2 h, which explains why singing activity resumed 125 min later but the quality of the songs produced at that time was not affected. If GABA_A_ activity was still increased when singing activity recovered, this would mean that PAG does not control song quality, since song was normal at that time. However, we do not believe that this interpretation is very likely, because it would then be difficult to understand why singing activity recovered.

In mammalian species, electrical stimulation of the PAG has been shown to elicit innate, species-specific calls (Jürgens, 1994). However, little is known concerning the function of PAG in regulating learned vocalizations, such as song. Projections from the PAG to HVC have been found to be important during zebra finch (*Taeniopygia guttata*) development and song learning, allowing juveniles to detect the presence of a tutor and encode the tutor song (Tanaka et al., 2018). Our results indicate that the medial PAG has additional influence on adult song, presumably by transmitting signals from POM to the song system.

The nature of the information transferred from POM to PAG would however require additional investigations. In canaries, we have collected extensive evidence indicating that T action in the POM is necessary for singing behavior to occur but does not modify song quality (Riters and Ball, 1999; Alger and Riters, 2006; Alger et al., 2009; Alward et al., 2013, 2016a,b; Vandries et al., 2019). We have therefore hypothesized that the POM modulates the motivation to sing, but it could also be postulated that these effects reflect a modulation of the motor aspects of singing. It is challenging to distinguish between these two hypotheses. The term motor control is usually used for mechanisms that are relatively close to the effector muscles, including for example the motor magnocellular neurons directly projecting to the spinal chord or possibly neurons in the PAG. The preoptic area is rather considered as an integration area that modulates higher order processes, including motivation. It is often assumed in the literature on male sex behavior in rodents that an increased latency to show sexual behavior after introduction of a female reflects a decrease in motivation (Melis and Argiolas, 1995), and we think that the delay in singing initiation reflects here a similar mechanism. The interpretation of the present experiments is thus probably not that inactivating PAG directly blocks a motor transmission to the muscles of the syrinx (such a direct projection is not known to exist) but rather that this inactivation interrupts transmission of a “motivational” signal from POM to HVC and/or RA. It is however true that RA is myotopically organized based on the muscles of the syrinx (Vicario, 1991) so an indirect modulation of the motor control of the syrinx via the PAG projection to RA could potentially contribute to the effects we have observed in this study.

The observation that the general activity of the birds (i.e., perch hopping) and the rate of calling behavior were not affected by muscimol during the period when song was inhibited supports the specificity of the effect on song and argues against an interpretation that would be based on a general inhibition of activity or on a non-specific stress response. However, given the difficulties of ascribing a strictly motivational role to the observed effect of transiently inactivating PAG, additional data would be required to completely dissociate this region’s effects on motivation as compared with the motor control of the syrinx.

Notably, it appears that the medial PAG, and not ICo, regulates these motivational signals. Birds with cannulas targeting this region took an average of 125 min longer to begin singing after muscimol infusions, presumably refraining from singing until the muscimol wore off, while birds with cannulas targeting other regions did not display a large difference in song latency between muscimol and saline trials. Immunohistochemical analysis has indicated that this region, referred to as the mesencephalic central gray in some older publications (Stokes et al., 1974), is organized like a folded open ventral mammalian PAG, while the laterally adjacent ICo appears to be homologous to the dorsal mammalian PAG (Kingsbury et al., 2011). In addition, the medial PAG contains the A11 group of catecholaminergic neurons, sending projections to the song control system (Appeltants et al., 2000, 2002). We hypothesize that the motivational cues from the POM are transmitted to the song system via these projections. Future studies should investigate these catecholaminergic inputs into song control nuclei and their influence on song behavior.

These results indicate that PAG is involved in the initiation of singing behavior and may regulate the motivation to produce song, but cannulas misplaced in the ICo served as a valuable control as ICo has been implicated in production of innate calls (Popa and Popa, 1933; Nieder and Mooney, 2020). Because of this literature, we expected a decrease in calling behavior following muscimol infusion into this region. However, we did not observe such a difference, likely due the placement of cannula. For cannulas targeting PAG, the medial position of the nucleus allowed muscimol to spread and to affect the bilateral extent of the structure. In contrast, when the cannula was located in ICo, a nucleus located more laterally, muscimol was not able to spread to the contralateral side to induce bilateral inactivation. Bilateral lesions of ICo decrease ring dove (*Streptopelia risoria*) nest coos, but only unilateral activation of this area with steroid hormones is required to induce an increase in nest-cooing behavior (Cohen and Cheng, 1981, 1982). Therefore, it is possible that using two cannulas to bilaterally inactivate this region would reduce calling behavior, a hypothesis that could be tested in future experiments. In addition, the cannulas classified as being in ICo in this study targeted the medial, rather than lateral, portion of ICo. Since many of the electrical stimulation studies implicating ICo as a regulator of calling behavior targeted the lateral region of the nucleus close to the dorsomedial nucleus of ICo (DM), it is more likely that this lateral portion is responsible for innate vocalizations (Seller, 1980). Therefore, targeting muscimol treatment in this lateral portion of ICo may potentially result in an inhibition of calling behavior.

Now that PAG has been identified as a region essential for the initiation of song, and potentially controlling the motivation to produce learned vocalizations, future research is required to identify the underlying cellular and molecular mechanisms. Systematically exploring how this region interacts with the song control system and the POM will advance our understanding of the biological underpinnings of social communication.

## Synthesis

Reviewing Editor: Rae Silver, Columbia University Decisions are customarily a result of the Reviewing Editor and the peer reviewers coming together and discussing their recommendations until a consensus is reached. When revisions are invited, a fact-based synthesis statement explaining their decision and outlining what is needed to prepare a revision will be listed below. The following reviewer(s) agreed to reveal their identity: jacob edward, Elizabeth Adkins-Regan.

Overview:

This manuscript addresses an important question regarding the role of brain regions outside of the forebrain “song system” in songbird singing behavior. Songbirds are a very large group of species, and are important subjects for research on brain mechanisms for learning of vocalizations and on forebrain mechanisms for learned skilled motor acts more generally. Brain regions outside the song system are almost certainly also involved in vocalization. Research on those brain regions may generalize to non-songbirds and perhaps even to other vertebrates. In the present study, the subjects are male canaries. The work shows that transient inactivation of the periaqueductal gray (PAG) via muscimol increases the bird’s latency to sing. This supports the hypothesis that inputs to the preoptic area from the PAG play an important role in mediating the regulation of the motivation to sing. This focus is well justified in the introduction.

A strength of the experiment is the use of muscimol, infused via cannulas aimed at the PAG, to temporarily inactivate the PAG. Among the advantages of this approach is that each bird can be its own control by interspersing muscimol treatment trials with saline trials. Cannula placement and spread of the muscimol were confirmed at the end of the study. Another good feature of the methods is the standardization of the males’ testosterone levels through castration plus administration of a testosterone implant.

The key result is that in trials with muscimol delivered to the PAG, males took much longer to start singing after infusion compared with saline trials or compared with muscimol trials of males where the cannulas missed the PAG. No other features of singing that were measured (including measures of song quality) were affected. The authors conclude that a functioning PAG is involved in the motivation to sing. Some cannula misses were located in the intercollicular nucleus, a structure identified in lesion studies to be involved in vocalization in other kinds of birds. Muscimol delivered there had no effect, and the discussion does a good job of explaining why that might have been the case.

The paper is clearly written and both the experiment and the study are straightforward.

Major comments:

Two issues need attention in order for the main conclusion of the study to be supported.

First, more than half the cannulas missed the PAG, leaving only four birds in the PAG group. The authors have done an ANOVA with the birds divided into three groups, PAG, ICO, and other misses. With 14 birds total, the numbers in each group are only 4-5, and in the figures the standard errors are quite variable in magnitude. I am concerned that assumptions for an ANOVA are being violated seriously. In the figure showing the key result (latency to start singing), the multiple muscimol trials and multiple saline trials have each been averaged and the two averages are shown. The reader can see that each of the four PAG birds did show a substantial increase in average latency following muscimol. With such a small N, this would be more compelling if all the trials were shown. Did each bird have a longer latency on each muscimol trial than on any saline trials?

eNeuro provides careful guidelines on the appropriate use of statistical analysis. The eneuro paper by Christophe Bernard should be consulted.

Second, there is a problem with the interpretations of the results as an effect on song motivation. In lines 53 and 72 of the introduction, the authors imply or predict that an effect on motivation would be reflected in the frequency/amount of singing: these were not affected in this study. Then, after the fact, authors equate latency to start singing with motivation. Ideally, song motivation would be defined so that its relation to any particular song measure was established a priori. The most parsimonious interpretation of the effect is that PAG is involved in the initiation of singing but once the bird has begun the PAG is no longer involved in song amount or quality. Why should we equate “initiation” with “motivation?” Perhaps the delay in “initiation” is a strictly motor problem. That would be equally interesting. Why is a motivation effect being implicated when the results did not support the motivation prediction at the end of the introduction?

Interpreting a higher latency to sing as reduced motivation seems like an overreach. Basically, an operational definition or clarification of what motivation means for songbirds would be useful. Second, the authors find that PAG inhibition increases latency to singing, but the experiments do not exclude alternative hypotheses as to why birds delay singing in isolation. For example, perhaps the PAG mediates a stress response to isolation (e.g. Berton et al. 2007), or influences overall levels of activity/arousal (e.g. Lu, Jhou, & Saper 2006). In order to justify the motivation hypothesis, additional experiments or data would be necessary. For example, the authors could conduct the same experiment, but place birds in the presence of a receptive female, or some social context where motivation is likely to vary. Alternatively, could the video recording data indicate whether birds were differentially active/aroused in muscimol vs. saline trials (e.g. time spent hopping vs. sitting, or some other measure)? Put directly, it is important to re-frame the conclusions in terms of the data: PAG inhibition leads to increased latency to sing when isolated. One might speculate on what this latency to sing means in terms of motivation (and alternative explanations; e.g. stress or arousal) in the intro and discussion, rather than interpret the data as reduced motivation per se.

As is, the bulk of the discussion is devoted to summarizing the study’s results and discussing the limitations of calling behavior, which is clearly not the focus of the study. Given that the majority of the paper is on motivated singing behavior, additional consideration of how PAG activity fits into this picture could be helpful.

This work uses muscimol, a GABAa agonist, to inhibit activity of the PAG prior to isolation and audio/video recording of singing behavior. It would be helpful to comment on the known half-life/metabolism rate of muscimol in as close a system as possible. If, when measuring song features in the hour after first singing, the muscimol is “presumed to have worn off", what are the meanings behind these measures, and is this a fair comparison given the working hypothesis? Is there prior reason to think brief inactivation of the PAG will disrupt crystallized song?

Figure 1 showing the placement of cannulae is useful, but a figure (perhaps a panel within this figure) with raw histological data showing fluorescent muscimol spread along with defined region borders (e.g. using Nissl, NeuN, or DAPI, etc) is essential to ascertain that the infusions were localized in the target region.

Finally, regarding the analyses, it is not clear what the “single value” described on line 151 refers to, and how it enters into your analyses. Is this a single value that reflects an aggregation of all acoustic features, or a single value for each acoustic feature that is averaged across all vocalizations in the hour after first singing? The RMS amplitude data is potentially interesting, but did the video recordings indicate that birds spent equal time near/facing the recording microphone? It is possible that reduced arousal led birds to sit away from/not facing the microphone. In addition, it appears that the denominator degrees of freedom differ between the latency analyses and song measures analyses (11 vs 8). Can you verify this? Did a different subset of individuals enter into these two analyses?

Minor points:

Line 257 - The discussion concludes with the phrase “the cognitive and biological underpinnings of social communication.” Cognition is biological so this needs rewording.

Line 36 - insert a comma after “Currently"

Line 51 - “increases song rate”: do you mean “singing rate”? Not clear if this refers to number of song bouts per unit time or decrease in gaps between song syllables/notes (i.e. a faster song)

Lines ∼59 - is there evidence that these specific dopaminergic projections from PAG reach POM?

Line 65 - could you briefly specify this statement on PAG DA markers and song production? E.g., “DA markers positively correlate with singing rates...”?

Line 68 and throughout - plural of “cannula” should be “cannulae” or “cannulas.”

Line 67, sentence starting with “We implanted...” - this sentence reads like the guide cannula led to high singing rates. You could split this sentence to something like “We castrated male canaries and implanted testosterone-releasing devices to ensure high singing rates. We then implanted guide cannulas to target the PAG for neurochemical manipulation.”

Line 69 - rather than “several", say the exact number of trials, or “alternating"

Lines 71-72 - it seems that frequency of singing is not your main measure of “motivation” (latency to singing is). Also, a specific prediction is given, but not a hypothesis. Could you clarify what the hypothesis is here that leads to this specific prediction?

Lines 94-95 - grammatically, “The cannula was lowered... The skin was sutured...” reads easier

Line 105 - was any solution infused during the “mock infusion”? Please clarify how this differs from the acclimation handling

Line 115 - the injection rate is useful to know; what was the total volume administered?

Lines 123-131 - the video and audio measurements of latency are not independent measures, because they came from the same source file. Were video and audio analyzed by separate investigators? Did you find any differences in the comparison - if so, that might be useful to know?

Line 131 - why was one hour chosen to be the cutoff length post-first song?

Line 133 - “...defined as vocalizations... “ do you mean “bouts of vocalizations”?

Line 141 - Did you fix and cryoprotect the animals/tissue? If so, briefly specify the fixation and cryoprotection media

Line 148 - this is the first mention of call data. Please specify above in the methods how this was collected/determined from the recordings, and justify (in intro and/or methods) why call data was worth collecting and what differentiates calls from song in the current context

Lines 185-187 - because these values are not reported in a figure, please give some measure of variation (SE or SD) here

Line 205 - as above, I suggest replacing “decreases motivation” with “increases latency"

Lines 216-218 - it is appropriate to temper the interpretation of motivation

Line 220 - replace “this region", with “the medial PAG” for clarity

Line 220 - “took much longer” - could you use your data to make a stronger statement here? E.g. “took upwards of two hours longer"

Line 226 - “ICo appears to be homologous"

Line 230 - “motivational cues from the POM"; it seems the thesis of this paper is that the PAG is the source of motivational cues? As above, it seems difficult to define which brain regions are motivational vs purely sensory or motor.
